# Green Approach for the Effective Reduction of Graphene Oxide Using *Salvadora persica* L. Root (Miswak) Extract

**DOI:** 10.1186/s11671-015-0987-z

**Published:** 2015-07-03

**Authors:** Mujeeb Khan, Abdulhadi H Al-Marri, Merajuddin Khan, Mohammed Rafi Shaik, Nils Mohri, Syed Farooq Adil, Mufsir Kuniyil, Hamad Z Alkhathlan, Abdulrahman Al-Warthan, Wolfgang Tremel, Muhammad Nawaz Tahir, Mohammed Rafiq H Siddiqui

**Affiliations:** Department of Chemistry, College of Science, King Saud University, P.O. 2455, Riyadh, 11451 Kingdom of Saudi Arabia; Institute for Inorganic and Analytical Chemistry, University of Mainz, Duesbergweg 10-14, 55128 Mainz, Germany

**Keywords:** Graphene, Graphene oxide, Natural product, Green chemistry, Spectroscopy

## Abstract

Recently, green reduction of graphene oxide (GRO) using various natural materials, including plant extracts, has drawn significant attention among the scientific community. These methods are sustainable, low cost, and are more environmentally friendly than other standard methods of reduction. Herein, we report a facile and eco-friendly method for the bioreduction of GRO using *Salvadora persica* L. (*S. persica* L.) roots (miswak) extract as a bioreductant. The as-prepared highly reduced graphene oxide (SP-HRG) was characterized using powder X-ray diffraction (XRD), ultraviolet-visible (UV-vis) spectroscopy, Fourier transform infrared spectroscopy (FT-IR), Raman spectroscopy, X-ray photoelectron (XPS) spectroscopy, and transmission electron microscopy (TEM). Various results have confirmed that the biomolecules present in the root extract of miswak not only act as a bioreductant but also functionalize the surface of SP-HRG by acting as a capping ligand to stabilize it in water and other solvents. The dispersion quality of SP-HRG in deionized water was investigated in detail by preparing different samples of SP-HRG with increasing concentration of root extract. Furthermore, the dispersibility of SP-HRG was also compared with chemically reduced graphene oxide (CRG). The developed eco-friendly method for the reduction of GRO could provide a better substitute for a large-scale production of dispersant-free graphene and graphene-based materials for various applications in both technological and biological fields such as electronics, nanomedicine, and bionic materials.

## Background

Among various carbonaceous materials, graphene has attracted tremendous attention of scientists and technologists, due to its stable 2D morphology and exceptional electronic properties related to its crystal structure [1–3]. Indeed, graphene has revolutionized the field of nanotechnology and has emerged as a promising new nanomaterial for a variety of exciting applications, despite being discovered recently. Remarkable thermal, electrical, and mechanical properties of graphene have been extensively exploited in various fields, including sensors [4], solar cells [5], nanoelectronics [6], energy storage [7], functional nanocomposites [8], biomedicine [9], and catalysis [10, 11]. Commonly, graphene is obtained from graphite [12], which is a naturally occurring material and has been in use for centuries [13]. The free-standing single-layer of graphene was first obtained in 2004 by the isolation of graphene from graphite via micromechanical cleavage [14]. This fascinating approach of peeling off graphene layers from graphite can only be useful for fundamental science, and is not suitable for the large-scale production of graphene [15]. Therefore, tremendous attention is being paid to explore various alternative approaches for the low-cost and bulk production of graphene.

Several methods, including chemical vapor deposition (CVD) [16], arc discharge [17], epitaxial growth on SiC [18], chemical conversion, liquid phase exfoliation, and sequential oxidation reduction of graphite, have been reported [19]. Among all these methods, sequential oxidation and reduction of graphite has attracted significant attention, because it is benign, less expensive, and is more suitable for the bulk production of graphene [20, 21]. Although, the flakes of graphene-like sheets obtained from such methods are not defect free, these nanosheets are highly processable, described as highly reduced graphene oxide (HRG), and have been extensively applied for the preparation of various graphene-based functional bio- and nanocomposites [22].

Among various reduction methods (thermal, electrochemical, or chemical) [23–25] of graphite oxide (GO) or graphene oxide (GRO), chemical reduction is the most promising method and is extensively applied for the large-scale production of HRG [26]. Several reducing agents, such as hydrazine [27], ammonia borane complex [28], sodium hydride [29], and hydrohalic acid [30], have been used for the reduction of GRO to obtain HRG [31]. However, despite several advantages, the chemically reduced HRG has limited applications, as it tends to agglomerate strongly due to interlayer attractive van der Waals forces [32]. Therefore, further chemical stabilizers, such as porphyrins and pyrenebutyric acid, are frequently required to prevent these kinds of agglomerations [33]. Majority of the chemicals involved in the reduction and functionalization of GRO are highly toxic in nature, hazardous, and harmful to both environment and human life [34]. In addition, the presence of trace amount of highly toxic reducing agents on the surface of HRG could seriously alter several properties of HRG and has adverse effects on its biological applications [35].

However, in comparison to chemical reduction, the green reduction of GRO involves biocompatible ingredients under physiological conditions of temperature and pressure [36, 37]. To date, several green reductants extracted from microorganisms, marine organisms, or plant extracts have been applied for the preparation of HRG, including gallic acid, fluorescent protein, melatonine, ascorbic acid, and wild carrot roots [38–42]. Among these green reductants, plant extracts have been significantly exploited due to their low cost, bulk availability, and biocompatibility [43–46]. Recently, extracts of various plants were used both as reducing and stabilizing agents during the preparation of metallic nanoparticles and in some cases for the reduction of GRO [47–51].

In this study, we have applied *Salvadora persica* (SP) L. (miswak) extract as a bioreductant. ‘Miswak’ is an Arabic word which literally means ‘tooth-cleaning stick or chewing stick’ [52]. It is widely used as an oral hygiene tool in most parts of the world. Approximately, 182 plant species have been used as chewing sticks; however, roots of *S. persica* L. are the most common one and frequently used for this purpose. *Salvadora persica* L. is a glabrous tree or shrub belonging to family *Salvadoraceae* [53–55]. *S. persica* L. is found in many parts of the world. In Saudi Arabia; it is very widely spread, especially in the southern regions of Saudi Arabia. Because of the Sunnah (practices of prophet Muhammad^PBUH^) of prophet of Islam Muhammad^PBUH^, use of miswak obtained from roots of *S. persica* L. is very common and popular in the Muslim world. Besides having several benefits for oral hygiene, various parts of *S. persica* L. are also used in folk medicine for the treatment of several diseases. For example, it is reported to have diuretic, antiscorbutic, anthelmintic, and analgesic properties [56].

**Scheme 1:**
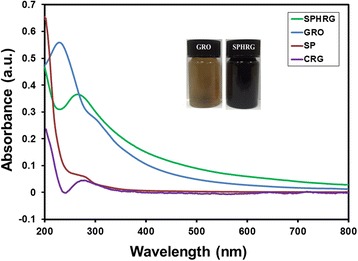
UV-vis absorption spectra of graphene oxide (GRO, *blue line*), highly reduced graphene oxide (SP-HRG, *green line*) reduced with PE, chemically reduced graphene oxide (CRG, *purple line*) and pure root extract (SP, *red line*). Although the concentration of CRG was the same as that of SP-HRG, it exhibits a much lower absorption coefficient due to its poor dispersibility in water

The entire plant of *S. persica* L. is considered to be edible, and it is reported that leaves of the plant are sometimes used in salad [57]. *S. persica* L. has been reported to have several important biological activities, such as antimicrobial, cytotoxic, hypoglycemic, antiulcer, antiplasmodial, and anti-inflammatory activities. Moreover, various classes of phytomolecules including terpenoids, alkaloids, flavonoids, saponins, and tannins have been isolated from this plant [56]. Thorough and detailed literature survey revealed that the use of *S. persica* L. as a bioreducing agent for the synthesis of nanomaterials has not yet been reported in literature. Thus, as part of our ongoing research on Saudi Arabian plants [58–60], we reported the green synthesis of HRG via the reduction of GRO using *S. persica* L. root (miswak) extract (cf. Scheme 1). The SP-HRG was characterized using various microscopic and analytical techniques including X-ray diffraction (XRD), Fourier transform infrared spectroscopy (FT-IR), ultraviolet-visible (UV-vis) spectroscopy, and transmission electron microscopy (TEM). During this study, a detailed analysis on the effect of plant extract (PE) on the dispersibility of HRG in various solvents was investigated by preparing HRG with different amounts of PE, and the dispersibility of HRG was compared with that of chemically reduced graphene oxide (CRG).

## Methods

### Materials

The roots of *S. persica* L. growing in Jizan, Southern region of Saudi Arabia were purchased from a local herbal market at Batha, Riyadh, Saudi Arabia. The identity of the plant material was confirmed by a plant taxonomist from the Herbarium Division of the College of Science, King Saud University, Riyadh, Saudi Arabia. A voucher specimen was retained in our laboratory with the voucher specimen number KSUHZK-302. Graphite powder (99.999 %, _200 mesh) was purchased from Alfa Aesar (USA). Concentrated sulfuric acid (H_2_SO_4_ 98 %), potassium permanganate (KMnO_4_ 99 %), sodium nitrate (NaNO_3_ 99 %) and hydrogen peroxide (H_2_O_2_ 30 wt %) and all organic solvents were obtained from Aldrich Chemicals (USA) and used without further purification.

### Preparation of *S. Persica* L. Root (Miswak) Extract

First, fresh roots of *S. persica* L. were cut into small pieces. The resultant pieces (1.3 kg) were soaked in deionized water (3000 mL) and refluxed for 4 h. Then, the aqueous solution obtained after reflux was filtered and dried at 50 °C under reduced pressure in a rotary evaporator to give a dark brownish gummy extract (30.0 g) which was stored at 0–4 °C for further use.

### Preparation of Highly Reduced Graphene Oxide (SP-HRG)

Graphite oxide (GO) required for the preparation of SP-HRG was synthesized according to our previously reported method [61]. Initially, as-prepared graphite oxide or GO (200 mg) was dispersed in 40 mL of DI water and sonicated for 30 min to obtain graphene oxide (GRO) sheets. The resulting suspension was taken in a round bottom flask mounted with a cooling condenser, which is heated to 100 °C. Subsequently, 10 mL of an aqueous solution of the root extract (0.1 g mL^−1^) was added to the suspension, which was then allowed to stir for 24 h at 98 °C. After this, black powder of highly reduced graphene oxide (PE-HRG-1) was collected by filtration, which was further washed with DI water several times to remove the excess root extract residue and redistributed into water for sonication. This suspension was centrifuged at 4000 rpm for another 30 min. Subsequently, the supernatant was thrown out and the precipitate was collected and dried in vacuum.

### Characterization

#### UV-vis Spectroscopy

A PerkinElmer lambda 35 (USA) UV-vis spectrophotometer was used for the optical measurements. The analysis was performed in quartz cuvettes using DI water as a reference solvent. Stock solutions of SP-HRG and GRO for the UV measurements were prepared by dispersing 5 mg of sample in 10 mL of DI water and sonicating for 30 min. The UV samples of GRO and SP-HRG were prepared by diluting 1 mL of stock solution with 9 mL of water.

#### X-ray Diffraction

XRD diffractograms were collected on a Altima IV (Rigaku, Japan) X-ray powder diffractometer using Cu Kα radiation (*λ* = 1.5418 °A).

#### Transmission Electron Microscopy

TEM was performed on a JEOL JEM 1101 (USA) transmission electron microscope. The samples for TEM were prepared by placing a drop of primary sample on a holey carbon copper grid and drying for 6 h at 80 °C in an oven.

#### Fourier Transform Infrared Spectrometer

FT-IR spectra were measured on a PerkinElmer 1000 (USA) Fourier transform infrared spectrometer. In order to remove any free biomass residue or unbound extract to the surfaces of SP-HRG sheets, the SP-HRG nanosheets were repeatedly washed with distilled water, and then the product was centrifuged at 9000 rpm for 30 min and dried. The purified SP-HRG nanosheets were mixed with KBr powder and pressed into a pellet for measurement. Background correction was made using a reference blank KBr pellet.

#### X-ray Photoelectron Spectroscopy

XPS spectra were measured on a PHI 5600 Multi-Technique XPS (Physical Electronics, Lake Drive East, Chanhassen, MN) using monochromatized Al Kα at 1486.6 eV. Peak fitting was performed using the CASA XPS Version 2.3.14 software.

## Results and Discussion

Reduction of GRO was carried out at an elevated temperature under reflux conditions using the root extract of miswak. Upon completion of reduction process, the brown color of GRO dispersion changed to dark black, which indicated the formation of SP-HRG. It is worth mentioning that no color change was observed in the absence of miswak root extract, under similar conditions. Although, 10 mL aqueous root extract (100 mg/mL) was found to be sufficient for the complete reduction of GRO, three different samples of SP-HRG (prepared by increasing the concentration of root extract) were also used to further investigate the effect of concentration of root extract on the dispersion quality of SP-HRG. The dispersion quality of the as-prepared samples of SP-HRG was also compared with chemically reduced GRO (CRG) using hydrazine as a reducing agent. The samples of SP-HRG were synthesized by using 10 mL (100 mg/mL) (SP-HRG-1), 20 mL (SP-HRG-2), and 50 mL (SP-HRG-3) of miswak root extract with concentration (100 mg/mL) while the amount of GRO was kept constant.

**Fig. 1:**
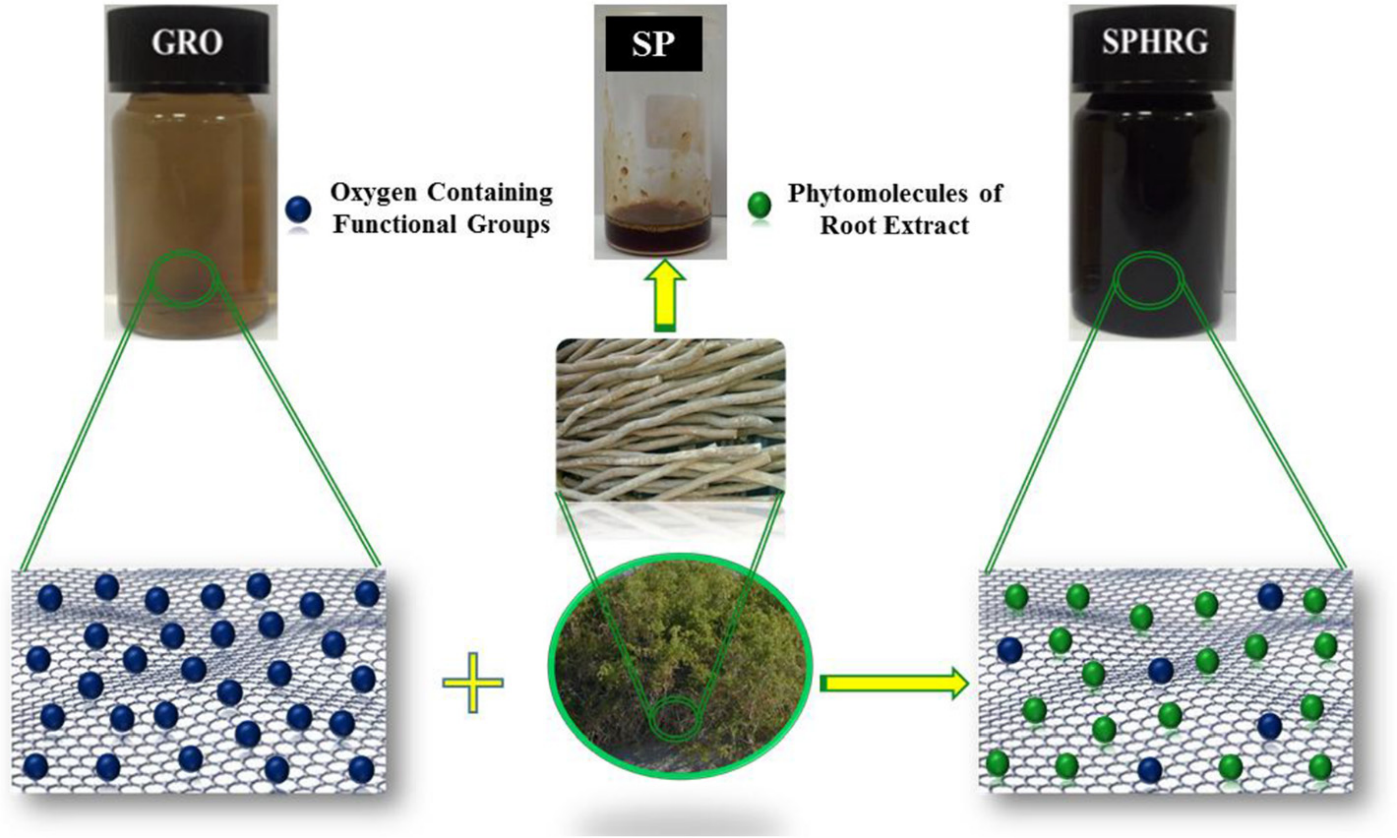
Schematic illustration of the green reduction of graphene oxide (GRO) using *Salvadora persica* L. (*S. persica* L.) roots (miswak) extract as a bioreductant

GRO reduction was initially monitored by recording UV-vis absorption spectra of both GRO and SP-HRG as shown in Fig. 1. GRO exhibited absorption peak at 230 nm which was attributed to the *π*–π* transitions of the aromatic C–C bonds and a weak shoulder at 301 nm due to *n*–π* transitions of C=O bonds present in GRO. However, on reduction of GRO to HRG, the characteristic absorption band at 301 nm disappeared and the absorption band at 230 nm in GRO spectrum is redshifted to 280 in SP-HRG, which confirms the reduction of GRO and the partial restoration of *π* network of SP-HRG. The absorption maximum in case of CRG appeared at 271 nm instead of 280 nm, which is considerably lower than that of SP-HRG. The higher redshift in case of SP-HRG is attributed to the superior degree of reduction in SP-HRG, as compared to CRG [61].

**Fig. 2:**
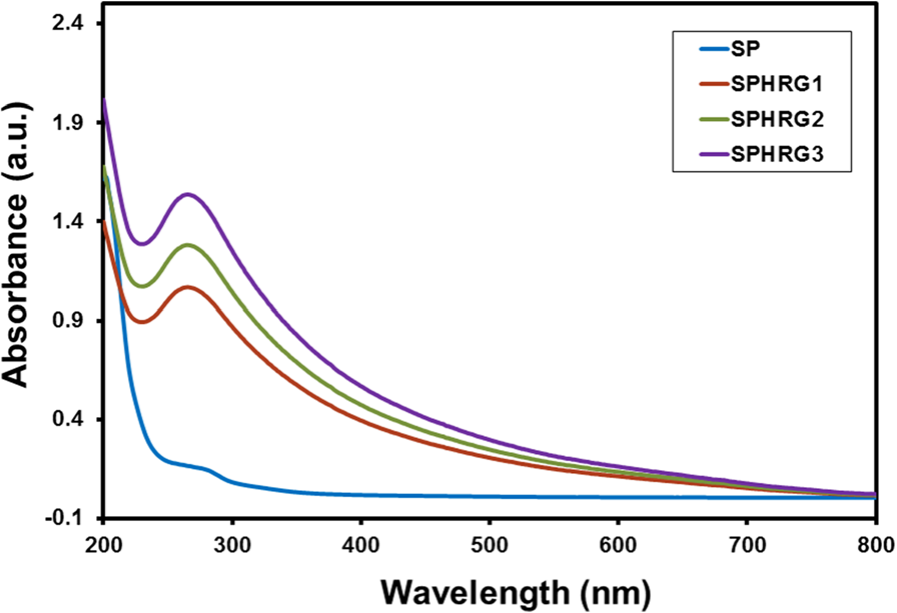
UV-vis absorption spectra of pure root extract (SP, *blue line*), SP-HRG-1 prepared with 10 mL root extract (*red line*), SP-HRG-2 prepared with 20 mL root extract (*green line*), and SP-HRG-3 prepared with 50 mL root extract (*purple line*)

The phytomolecules of miswak root extract not only reduce the GRO but also functionalize the surface of SP-HRG. This was confirmed in our previous study by the UV analysis of HRG obtained via reduction of GRO using *Pulicaria glutinosa* plant extract [61]. In order to investigate this, the UV spectrum of pure miswak root extract was measured, where the absorption maximum appeared at ~282 nm, which overlapped with the characteristic peak of SP-HRG (~280 nm). However, on further increasing the amount of plant extract and keeping the GRO amount constant, notably, the intensity of the absorption band at ~282 nm in these samples also increased (cf. Fig. 2). This clearly indicates the presence of phytomolecules of miswak root extract on the surface of SP-HRG.

**Fig. 3:**
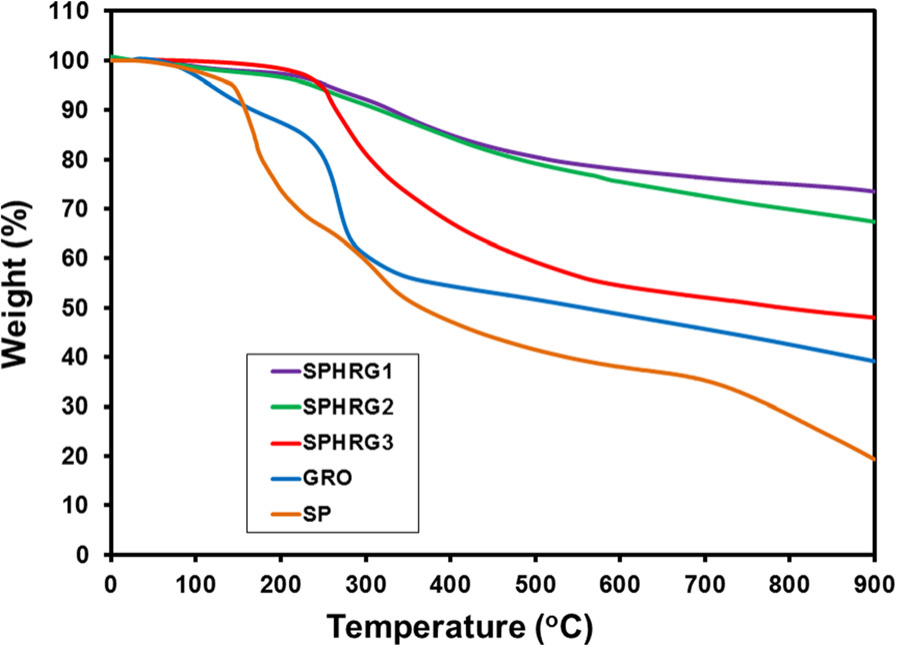
TGA traces of root extract (SP), graphene oxide (GRO), SP-HRG-1, SP-HRG-2, and SP-HRG-3

Thermal stability of GRO, SP-HRG-1, SP-HRG-2, SP-HRG-3, and miswak root extract were evaluated via TGA (cf. Fig. 3). Pure graphite does not exhibit any weight loss in the temperature range of 0–900 °C; however, GRO exhibited significant weight loss in several steps, due to the presence of various oxygen-containing functional groups [61]. The degradation of GRO involves two prominent steps, initial and relatively faster weight loss of up to ~40 % occurs between 100 and 300 °C, due to the loss of adsorbed water and labile oxygen-containing functional groups, such as hydroxyl and epoxy functional groups. Subsequently, in the second step, a gradual weight loss of ~20 % was observed between 300 and 900 °C (cyan line in Fig. 3), which is attributed to the pyrolysis of remaining oxygen-containing functional groups and burning of carbon skeleton [61].

Similarly, pure miswak root extract also exhibited up to ~80 % rapid weight loss between 100 and 900 °C in various steps (orange line in Fig. 3). However, SP-HRG exhibited only ~12–15 % weight loss between this temperature range, which was much lower than that of pure GRO and miswak root extract (purple line in Fig. 3). This is attributed to the significant decrease of oxygen-containing functional groups, which clearly indicates the reduction of GRO. Notably, with increasing concentration of miswak root extract, the weight loss of SP-HRG also increased, for instance, SP-HRG-1 showed a weight loss of ~12–15 % (purple line), SP-HRG-2 exhibited 22–25 % (green line), where as in SP-HRG-3 up to ~45 % of weight loss was observed (red line). This points towards the presence of phytomolecules of miswak root extract on the surface of SP-HRG.

**Fig. 4:**
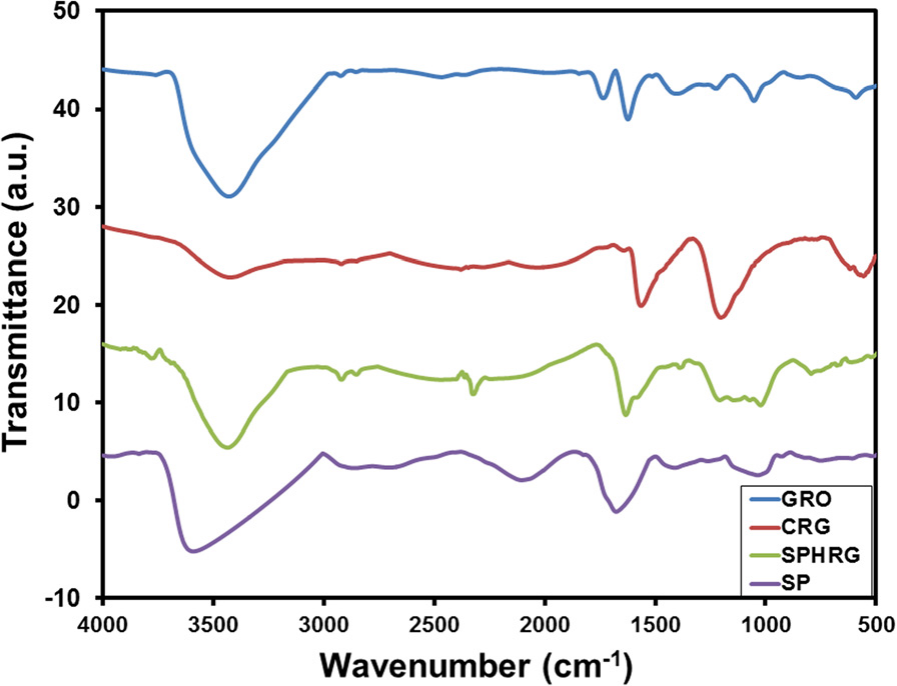
FT-IR spectra of graphene oxide (GRO, *blue line*), root extract-mediated highly reduced graphene oxide (SP-HRG, *green line*), and chemically reduced graphene oxide (CRG, *red line*) prepared with hydrazine hydrate and the pure root extract (SP, *purple line*)

The dual role of the miswak root extract as a bioreductant and capping agent was also confirmed by FT-IR analysis (cf. Fig. 4). FT-IR spectra of GRO (blue line), pure miswak root extract (purple line), SP-HRG (green line), and CRG (red line) were measured. The presence of intense bands at ~1740 cm^−1^ (for C=O stretching), ~1630 cm^−1^ (for C=C stretching), ~1209 cm^−1^ (for C–O–C stretching), ~1050 cm^−1^ (for C–O stretching), and a broad band at around 3440 cm^−1^ for hydroxyl groups indicated the presence of various oxygen-containing functional groups, such as carbonyl, carboxylic, epoxy, and hydroxyl groups in GRO. The removal of such oxygen-containing groups of GRO in the samples of SP-HRG and CRG was clearly indicated by the disappearance of some of the bands in their respective FT-IR spectra, such as the band at ~1740 and ~1630 cm^−1^. Also the relative decrease in the intensity of some of the other bands, like the decrease in intensity of broad band at 3440 cm^−1^ belonging to the hydroxyl groups of GRO, points towards the reduction of GRO. A relatively small decrease in the intensity of FT-IR signals in case of SP-HRG, when compared to CRG and also the presence of some additional bands in its IR spectrum, is attributed to the phytomolecules bound to the surface of SP-HRG post in-situ functionalization. This was further confirmed by comparing the IR spectra of SP-HRG and the pure miswak root extract. Most of the absorption bands of the miswak root extract also appear in the FT-IR spectrum of SP-HRG, either at same position or with slight shifts, such as the bands at ~3790, ~2329, 1636, and 1005 cm^−1^. The appearance of these bands in SP-HRG and their absence in CRG spectrum strongly suggests that the phytomolecules of miswak root extract act not only as bioreductant but also as stabilizers on the surface of the SP-HRG sheets.

**Fig. 5:**
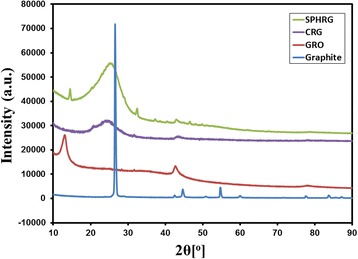
XRD diffractograms of graphite, graphene oxide (GRO), root extract-mediated highly reduced graphene oxide (SP-HRG), and chemically reduced graphene oxide (CRG)

Furthermore, the reduction of GRO was also monitored using XRD analysis. The XRD patterns of pristine graphite (blue line), GRO (red line), CRG (purple line), and SP-HRG (green line) are shown in Fig. 5. The pristine graphite exhibits a very intense and narrow reflection at 2*θ* = 26.4°, which is considerably shifted to lower Bragg angles at 2*θ* = 10.9° and slightly broadened in GRO, due to the formation and intercalation of oxygen-containing functional groups between the layers of carbon nanosheets. However, after the reduction, the reflection of GRO at 10.9° disappeared in both SP-HRG and CRG, and their interlayer distance also decreased due to the removal of oxygen-containing functional groups. Moreover, a broad reflection centered at 2*θ* = 22.4° in their diffraction patterns indicated the formation of graphene nanosheets with a thickness of few layers [30].

**Fig. 6:**
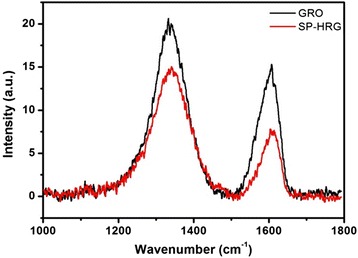
Raman spectra of graphene oxide (GRO) and highly reduced graphene oxide (SP-HRG) using root extract

The Raman spectra of graphene comprise two main features: the G and D bands at 1575 and 1350 cm^−1^, respectively. Figure 6 demonstrates the Raman spectra of GRO and SP-HRG. The G and the D bands of GRO are shifted and appear at 1602 and 1340 cm^−1^, respectively, due to the destruction of the *sp*^2^ character and the formation of defects in the sheets caused by the extensive oxidation. However after reduction, the G band in SP-HRG is slightly narrower and shifted to 1592 cm^−1^, and the D band is centered at 1336 cm^−1^. A comparison of the Raman spectra of both GRO and SP-HRG showed that the G band of SP-HRG is shifted by 10 cm^−1^ from 1602 to 1592 cm^−1^, whereas a slight shift was observed in the D band from 1340 to 1336 cm^−1^. The shifts in the bands of SP-HRG after the reduction towards the ideal positions of the G band (1575 cm^−1^) and D band (1350 cm^−1^) of graphene is a clear indication towards the restoration of the *sp*^2^ character of SP-HRG, and it is well-suited with a high degree of reduction.

**Fig. 7:**
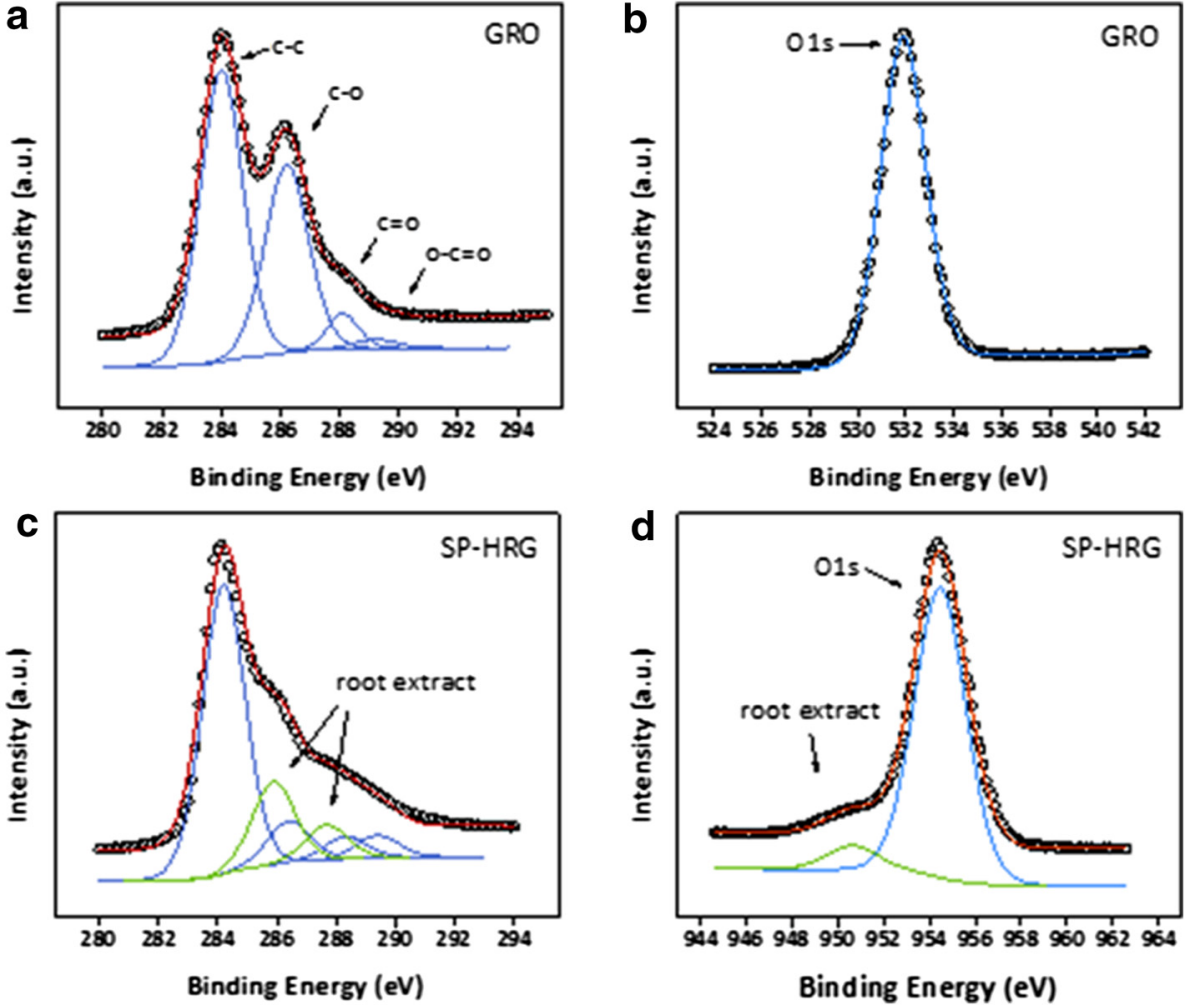
XPS spectra of graphene oxide (GRO): C1s (**a**) and O1s peak (**b**) and highly reduced graphene oxide with root extract (SP-HRG): C1s (**c**) and O1s peak (**d**)

Figure 7 shows a comparison of the XPS spectra of GRO and SP-HRG. The graphene sample (GRO) exhibits the typical peaks at 284.0, 286.2, 288.0, and 289.2 eV resulting from *sp*^3^ and *sp*^2^ C–C, C–O, C=O, and O–C=O groups, respectively (cf. Fig. 7a) [62]. Applying the same boundary conditions to the fit of the reduced sample (SP-HRG) results in a deviation at 285.9 and 287.7 eV originating from C–C and C–O groups of the root extract (cf. Fig. 7c). The additional shoulder in the O1s peak of SP-HRG (cf. Fig. 7d), which is missing in the GRO spectrum (cf. Fig. 7b), confirms the presence of root extract residues on the surface of the sample. Calculation of the atomic concentrations shows the amount of C–O and C=O groups on SP-HRG to be reduced significantly (35.74 to 7.11 % and 6.83 to 4.33 %, respectively), while maintaining the amount of C–C groups (56.24 to 57.83 %), representing the high degree of reduction on the surface of the sample. The peak at 289.2 eV increases after reduction (1.19 to 5.14 %), which points to an additional peak originating from C=O groups of the plant extract, which was not added due to the low intensity.

**Fig. 8:**
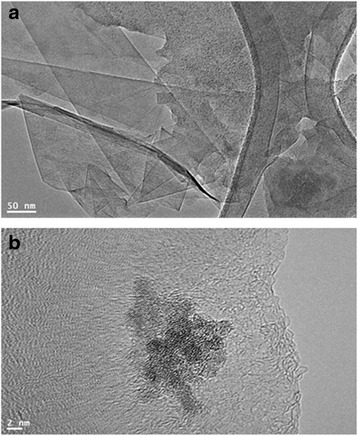
TEM images (**a** and **b**) of as-prepared SP-HRG at different resolutions

Figure 8a, b illustrates the morphology and layer thickness of the SP-HRG, which were determined by transmission electron microscopy (TEM). The TEM images revealed the transparent and sheet-like structure of SP-HRG. An enormous number of scrolls and wrinkles were observed on the surface of the SP-HRG sheet, which remained stable under the high-energy electron beam. It has been noticed that the edges of the suspended graphene layers were folded back, and few layer thickness of SP-HRG was observed in the high-resolution TEM.

The large-scale production of graphene obtained via solution-based sequential oxidation-reduction processes usually suffer from poor dispersibility in water and other organic solvents, due to their strong hydrophobic nature [63]. The irreversible agglomeration of graphene nanosheets is usually prevented by the addition of various external surfactants and stabilizers, including polymers and dendrimers [64], which have undesirable effects on the properties of graphene. However, during the green reduction of GRO using plants extracts, additional surfactants or stabilizers are not required, wherein the plant extracts themselves act as both reducing as well as stabilizing agents [61]. The dispersion quality of HRG obtained using root extract of miswak (SP-HRG) is investigated and also compared with chemically obtained HRG (CRG). For this purpose, various samples of SP-HRG are prepared by increasing the concentration of miswak root extract. For instance, SP-HRG-1, SP-HRG-2, and SP-HRG-3 are prepared by using 10, 20, and 50 mL of miswak root extract (100 mg/mL), respectively.

**Fig. 9:**
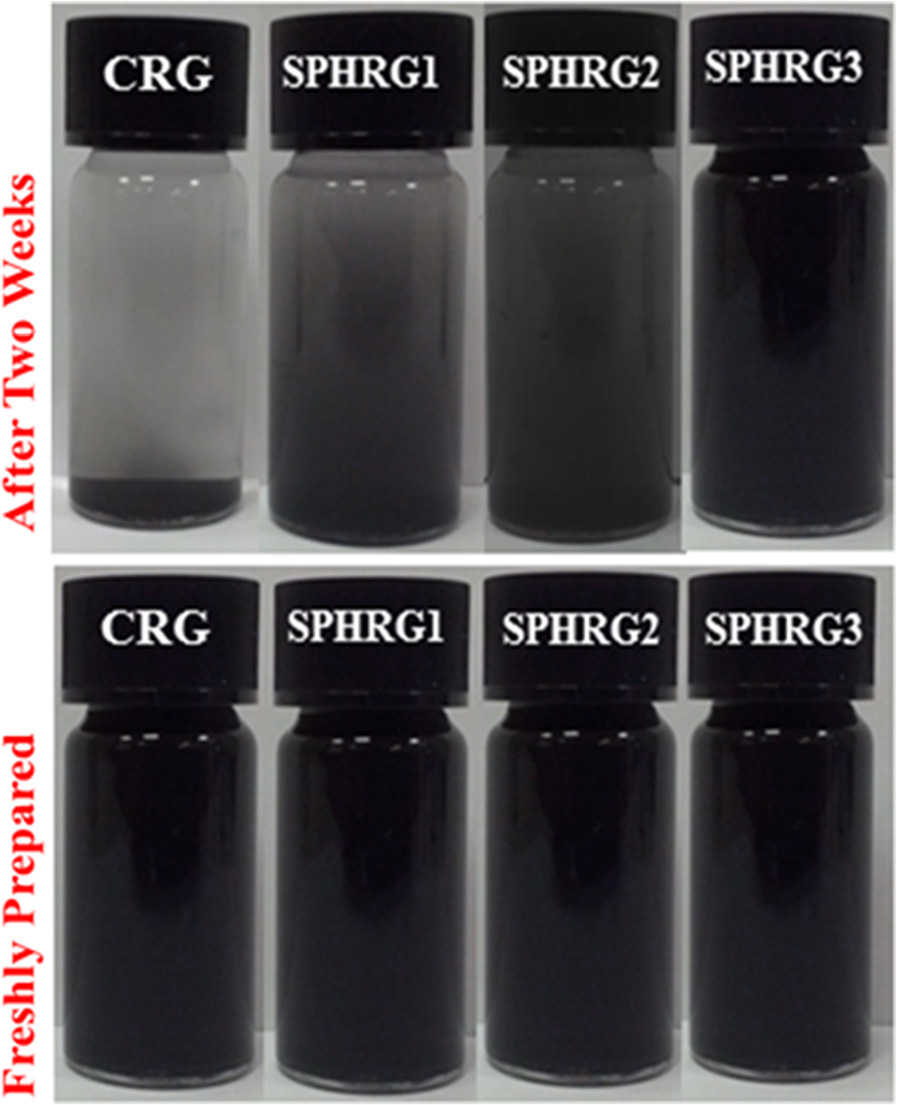
Digital images of the dispersions of CRG, SP-HRG-1 prepared with 10 mL of plant extract, SP-HRG-2 prepared with 20 mL of plant extract, and SP-HRG-3 prepared with 50 mL of plant extract and CRG (prepared with hydrazine hydrate)

The dispersions of these samples and CRG are prepared by sonicating 5 mg of each sample in 10 mL of DI water. Superior dispersions were obtained for the bioreduced SP-HRG as compared to CRG. Notably, the dispersion quality of SP-HRG samples improved with increasing the concentration of miswak root extract. For example, SP-HRG-3 demonstrated an excellent dispersion, which remained stable even after 2 weeks compared to relatively lower stability SP-HRG-1. However, after 2 weeks, the CRG suspension became completely unstable, whereas all the samples of SP-HRG demonstrated excellent dispersibility in water as shown in Fig. 9.

## Conclusions

Graphene oxide is reduced using an eco-friendly route, i.e., root extract of *S. persica* (miswak) as both a bioreductant and stabilizer. The as-prepared bioreduced SP-HRG exhibited excellent dispersibility compared to the chemically reduced graphene oxide (CRG). The concentration of *S. persica* (miswak) root extract played a critical role in the dispersibility of SP-HRG, whereas SP-HRG prepared using a high concentration of miswak root extract (SP-HRG-3) demonstrated superior dispersion in DI water. This clearly indicates that the miswak root extract acted not only as a bioreductant but also as a capping ligand, which was confirmed by various spectroscopic techniques. Therefore, the protocol presented here for the bioreduction of GRO can be potentially applied for the large-scale production of dispersant-free graphene nanosheets. Indeed, the highly oxidized nature, abundancy, and low cost of miswak root extract can be further exploited for the up-scaling of graphene and graphene-based materials for various technological and biological applications.
